# Clinical Predictors of Surgical Management in Patients with Chronic Epiphora: A CT-DCG–Supported Analysis

**DOI:** 10.3390/diagnostics16111740

**Published:** 2026-06-05

**Authors:** Akerke Makenkyzy, Zhanar Abdrakhmanova, Raushan Rakhimzhanova, Zeynet Akhmedyanova, Elvira Kadralieva, Aigerim Tuletova, Zhibek Dautbayeva

**Affiliations:** 1 Department of Eye Diseases, NJSC “Astana Medical University”, Astana 010000, Kazakhstan; akerke.makenkyzy2025@gmail.com (A.M.); ahmedyanova.z@amu.kz (Z.A.);; 2 Department of Radiology, NJSC “Astana Medical University”, Astana 010000, Kazakhstan; 3 Kazakh Eye Research Institute, Astana 010000, Kazakhstan

**Keywords:** epiphora, nasolacrimal duct obstruction, CT dacryocystography, lacrimal drainage system, surgical management, diagnostic imaging

## Abstract

**Background**: Chronic epiphora is a common ophthalmic complaint with multifactorial etiology, including both structural obstruction of the nasolacrimal drainage system (NLDS) and functional or ocular surface–related causes. Accurate differentiation between these conditions is essential for appropriate management, yet conventional diagnostic methods provide limited anatomical detail. The complementary role of advanced imaging in routine clinical decision-making remains incompletely defined. **Methods**: In this retrospective observational study, 127 patients with chronic epiphora who underwent CT-DCG as part of routine clinical care were analyzed. CT-DCG was performed in patients with suspected NLDS involvement. Clinical, functional, and imaging data were analyzed, and multivariable logistic regression was used to identify factors associated with surgical management. **Results**: Distal NLDS involvement was the most common CT-DCG finding. Purulent discharge and longer symptom duration were associated with surgical management. Duration of symptoms remained independently associated with surgical management (OR 1.04 per month, 95% CI 1.02–1.06, *p* < 0.001), while purulent discharge was also strongly associated, although the confidence interval was wide. Functional parameters, including fluorescein dye disappearance test and dry eye syndrome, were not independently associated. The model demonstrated good discrimination (AUC 0.89). **Conclusions**: CT-DCG provides detailed anatomical assessment of lacrimal drainage abnormalities in patients with chronic epiphora and may complement standard clinical evaluation in selected cases with suspected structural pathology. However, CT-DCG findings were analyzed primarily descriptively because several imaging subgroups were small. Clinical features such as prolonged symptom duration and purulent discharge were associated with surgical management, whereas functional tests alone showed limited independent association.

## 1. Introduction

Chronic epiphora is a common presenting complaint in ophthalmic practice and has a heterogeneous, multifactorial etiology [1]. In addition to nasolacrimal drainage system (NLDS) obstruction, excessive tearing may result from ocular surface disease, eyelid malposition, and functional tear outflow abnormalities, which can complicate diagnosis and management [2,3]. The NLDS comprises the puncta, canaliculi, lacrimal sac, and nasolacrimal duct, and pathology may arise at different levels of this pathway [3,4]. Among structural causes in adults, primary acquired nasolacrimal duct obstruction (PANDO) is one of the most common lacrimal drainage disorders and is reported more frequently in women, particularly middle-aged and postmenopausal women; proposed explanations include anatomical differences in bony nasolacrimal duct dimensions and hormonal influences [5,6,7]. However, clinical examination alone may be insufficient to localize the level of obstruction precisely or to distinguish structural obstruction from functional epiphora, particularly in complex cases [8].

Traditional diagnostic methods, including lacrimal irrigation and fluorescein dye disappearance testing, provide useful functional information but limited anatomical detail [9]. As a result, they may not reliably define the precise site or extent of obstruction, especially in partial, functional, or otherwise complex cases [10]. This limitation has increased interest in imaging modalities that can improve anatomical localization and provide additional structural information in selected patients. Computed tomographic dacryocystography (CT-DCG) is a valuable imaging technique that provides high-resolution visualization of the lacrimal drainage system as well as adjacent bony and sinonasal structures [11]. By combining contrast enhancement with cross-sectional imaging, CT-DCG can localize stenosis or obstruction and identify associated anatomical abnormalities relevant to management. Previous studies have shown its usefulness in preoperative assessment, including recurrent obstruction, trauma-related disease, and failed dacryocystorhinostomy [8,10,11]. However, the role of CT-DCG in the routine evaluation of chronic epiphora remains incompletely defined, particularly with regard to distinguishing structural nasolacrimal obstruction from non-obstructive causes of tearing, which is important for patient selection and treatment planning.

The study aimed to evaluate CT-DCG findings in patients with chronic epiphora, with a particular focus on structural NLDS abnormalities and factors associated with surgical management.

## 2. Materials and Methods

### 2.1. Study Design and Setting

This single-center retrospective observational study analyzed clinical and imaging data obtained during routine ophthalmologic care between December 2022 and May 2024 at the Department of Ophthalmology and the Scientific Research Institute of Radiology, NJSC Astana Medical University (Astana, Kazakhstan). The study aimed to evaluate CT-DCG findings in patients with chronic epiphora, with a particular focus on structural NLDS abnormalities and factors associated with surgical management.

### 2.2. Study Population

**Figure 1:**
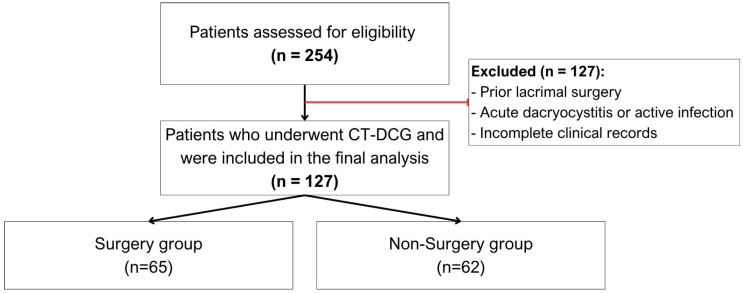
Flow diagram of patient selection and inclusion in the study.

A total of 254 adult patients presenting with unilateral or bilateral chronic epiphora lasting more than six months were assessed during the study period. CT-DCG was selectively performed in patients with clinical suspicion of NLDS involvement following initial ophthalmologic evaluation. This resulted in a subgroup of 127 patients who underwent CT-DCG and were included in the final analysis (Figure 1).

Patients were eligible for inclusion if they had persistent epiphora lasting more than six months and complete clinical and imaging records available for retrospective analysis. Patients were excluded if they had acute dacryocystitis or other active ocular infections, a history of prior lacrimal surgery including external or endoscopic dacryocystorhinostomy, known hypersensitivity to iodinated contrast agents, contraindications to CT imaging, or pregnancy or lactation. Consecutive patient records meeting the eligibility criteria were included to minimize selection bias. No formal sample size calculation was performed. The study size was determined by the number of eligible patients presenting during the study period who met the inclusion criteria and underwent CT-DCG as part of routine clinical evaluation.

### 2.3. Clinical Evaluation and Initial Classification

Clinical examination findings obtained during routine ophthalmologic evaluation were retrospectively reviewed. The assessment included slit-lamp biomicroscopy, evaluation of tear meniscus height, fluorescein dye disappearance testing, tear film breakup time (TBUT), and lacrimal irrigation using a lacrimal cannula attached to a 2 mL syringe to assess functional patency. Additional evaluation included assessment of eyelid position and stability (including ectropion and entropion), punctal status, trichiasis, meibomian gland dysfunction, pterygium, and signs of chronic blepharoconjunctivitis to identify non-obstructive or reflex causes of tearing. Some patients reported intermittent worsening of symptoms despite an overall chronic disease course. Based on recorded clinical findings, patients were categorized as having suspected ocular surface–related epiphora or suspected NLDS-related epiphora. CT-DCG was subsequently performed in patients with suspected NLDS involvement to confirm or exclude structural obstruction and to localize the level of pathology. All clinical assessments were performed using standardized methods and applied uniformly across all participants, irrespective of subsequent management strategy. Advanced tear meniscus imaging techniques, such as anterior segment optical coherence tomography (AS-OCT) were not routinely available during the study period.

The analysis was performed at the patient level rather than the eye level. In patients with bilateral epiphora, retrospective clinical and imaging assessment was based on the side considered more clinically significant according to the medical records.

### 2.4. CT-DCG Protocol

All CT-DCG examinations were performed using an 80-slice multislice CT scanner (United Imaging uCT 520, Shanghai United Imaging Healthcare Co., Ltd., Shanghai, China). CT-DCG examinations were performed as part of routine diagnostic evaluation and not specifically for research purposes. A non-contrast scan was first obtained to evaluate baseline orbital and sinonasal anatomy. Topical anesthesia was administered using 0.5% proparacaine hydrochloride (Alcaine^®^, Alcon Laboratories, Inc., Fort Worth, TX, USA). Under aseptic conditions, 1.0 mL of a water-soluble iodinated contrast agent (Omnipaque^®^, iohexol; GE HealthCare Ireland Limited, Cork, Ireland) was instilled into the lower lacrimal punctum using a lacrimal cannula. Spiral CT imaging was then performed in axial and coronal planes with a slice thickness of 1.0 mm and a reconstruction interval of 0.55 mm.

Images were evaluated by a radiologist and an ophthalmologist, with final interpretation reached by consensus. Structural obstruction was defined as complete absence of contrast passage or clear anatomical blockage at any level of the lacrimal drainage system, while partial stenosis was defined as delayed or reduced contrast passage associated with luminal narrowing. The level of obstruction was categorized as punctal, canalicular, common canalicular, lacrimal sac, or nasolacrimal duct. Additional findings, including sac distension, ductal ectasia, dacryocystocele formation, and sinonasal abnormalities, were recorded.

### 2.5. Outcome Measures

Decisions regarding surgical management were made during routine clinical care by the treating ophthalmologists based on the overall clinical presentation, including symptom severity and duration, ophthalmologic examination findings, lacrimal irrigation results, and CT-DCG findings when available. Surgical procedures included dacryocystorhinostomy and other lacrimal drainage interventions considered clinically appropriate for the identified pathology. No predefined surgical protocol or blinding procedures were applied due to the retrospective observational design of the study.

The primary outcome of the study was surgical management, defined as performance of a lacrimal drainage procedure based on combined clinical and imaging findings. CT-DCG findings were evaluated to characterize structural abnormalities of the nasolacrimal drainage system and to assess their relationship with surgical management.

### 2.6. Statistical Analysis

Statistical analysis was performed using GraphPad Prism version 10.4.1 (GraphPad Prism, Dotmatics, Boston, MA, USA) and Python version 3.10.12 (Python Software Foundation, Wilmington, DE, USA). Continuous variables were expressed as mean ± standard deviation or median with interquartile range, depending on data distribution, and categorical variables were presented as frequencies and percentages. Variables were selected based on clinical relevance and prior evidence of association with lacrimal drainage obstruction. CT-DCG findings were primarily analyzed descriptively because several imaging subgroups contained small numbers of patients, which limited their suitability for stable multivariable modeling. Comparisons between patients who underwent surgery and those managed conservatively were performed using the Mann–Whitney U test for continuous variables and Fisher’s exact test for categorical variables. Multivariable logistic regression analysis was conducted to identify independent predictors of surgical intervention. Variables included in the model were duration of symptoms, fluorescein dye disappearance score, presence of purulent discharge, ENT comorbidity, dry eye syndrome, and history of ocular trauma. Odds ratios (ORs) with 95% confidence intervals (CIs) were calculated, and statistical significance was defined as *p* < 0.05. Multicollinearity among predictors included in the logistic regression model was assessed using variance inflation factors (VIFs). Logistic regression model assumptions, including absence of severe multicollinearity and adequacy of model fit, were assessed using VIFs and the Hosmer–Lemeshow goodness-of-fit test.

To minimize selection bias, consecutive patient records obtained during the study period were included in the analysis. Clinical and imaging assessments had been performed according to standardized institutional protocols, and the resulting records were retrospectively reviewed to reduce measurement bias. Multivariable logistic regression was used to adjust for potential confounding factors. Only patients with complete clinical and imaging data were included in the final analysis, no imputation for missing data was required.

## 3. Results

### 3.1. Baseline Characteristics of the Study Population

A total of 127 patients who underwent CT-DCG were included in the analysis. Detailed baseline characteristics of the study population are presented in Table 1. The median age of the study population was 64 years (IQR 49.5–70), and the majority of patients were female (102, 80.3%). The median duration of symptoms was 26 months (IQR 9.5–53), indicating a predominantly long-standing clinical course. Dry eye syndrome was present in 87 patients (68.5%), while purulent discharge was observed in 52 patients (40.9%). ENT comorbidities were identified in 57 patients (44.9%), and a history of ocular trauma was reported in 20 patients (15.7%). Most patients demonstrated a persistent chronic course of epiphora (81, 63.8%), while 46 patients (36.2%) reported intermittent exacerbations of symptoms. Functional assessment showed a median fluorescein dye disappearance score of 2 (IQR 2–3). Tear production, as measured by the Schirmer test, demonstrated median values of 12 mm (IQR 10–16) in the right eye and 13 mm (IQR 11–16) in the left eye.

### 3.2. CT-DCG Findings

**Figure 2:**
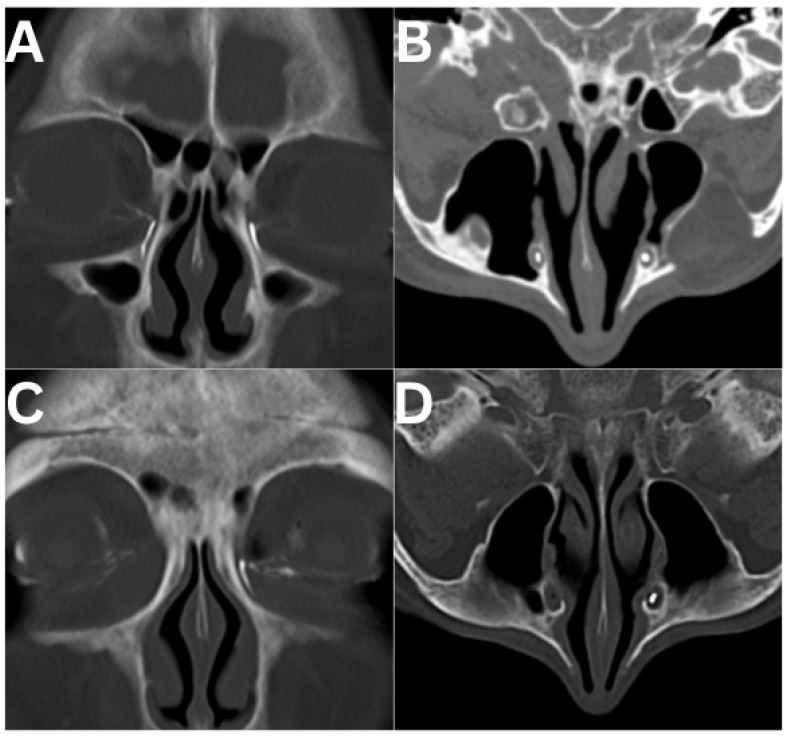
CT-dacryocystography findings in patients with chronic epiphora. (**A**,**B**) Normal nasolacrimal duct patency with continuous passage of contrast material into the nasal cavity. (**C**,**D**) Nasolacrimal duct obstruction demonstrating accumulation of contrast material with absence of distal flow.

CT-DCG revealed a heterogeneous spectrum of structural abnormalities of the lacrimal drainage system (Table 2, Figure 2). The most frequent finding was nasolacrimal duct stenosis, observed in 50 patients (39.4%), followed by complete nasolacrimal duct obstruction in 35 patients (27.6%). Together, these findings indicate that structural nasolacrimal duct pathology accounted for the majority of cases. Inflammatory conditions were also commonly identified, with dacryocystitis present in 21 patients (16.5%). Less frequent findings included punctal eversion in 10 patients (7.9%), canaliculitis in 6 patients (4.7%), dacryolithiasis in 3 patients (2.4%), and dacryopericystitis in 2 patients (1.6%). Overall, CT-DCG provided detailed anatomical characterization of lacrimal drainage abnormalities, demonstrating a predominance of distal nasolacrimal duct involvement in patients with chronic epiphora.

### 3.3. Comparison Between Surgical and Non-Surgical Management

Patients were stratified according to management strategy into surgical (*n* = 65) and non-surgical (*n* = 62) groups (Table 3). There were no significant differences between the groups in terms of age (median 65 vs. 59.5 years, *p* = 0.213) or sex distribution (84.4% vs. 75.8% female, *p* = 0.381). Similarly, Schirmer test values in both eyes and the history of ocular trauma did not differ significantly between the groups. The duration of symptoms was significantly longer in the surgical group compared to the non-surgical group (median 38 vs. 14 months, *p* < 0.001). In addition, the fluorescein dye disappearance score was significantly higher in the surgical group (median 3 vs. 2, *p* < 0.001), indicating more pronounced impairment of tear drainage. Purulent discharge was markedly more frequent among patients undergoing surgery (66.2% vs. 14.5%, *p* < 0.001) and was strongly associated with surgical management (OR 11.51, 95% CI 4.78–25.74). Dry eye syndrome was also more prevalent in the surgical group (80.0% vs. 56.5%, *p* = 0.007) and was associated with an increased likelihood of surgery (OR 3.09, 95% CI 1.43–6.83). In contrast, ENT comorbidities were more frequently observed in the non-surgical group (58.1% vs. 32.3%, *p* = 0.004) and were associated with a reduced likelihood of surgical intervention (OR 0.34, 95% CI 0.17–0.71). No statistically significant difference was observed in the distribution of intermittent symptom exacerbation between the groups (*p* = 0.052).

Analysis of CT-DCG findings demonstrated that most structural abnormalities were similarly distributed between the groups. NLDS stenosis and obstruction were the most common findings in both groups, without significant differences. However, punctal eversion was significantly more frequent in the surgical group (13.8% vs. 1.6%, *p* = 0.017). Other findings, including dacryocystitis, canaliculitis, dacryolith, and dacryopericystitis, did not differ significantly between the groups.

### 3.4. Multivariable Analysis of Predictors of Surgical Management

**Figure 3:**
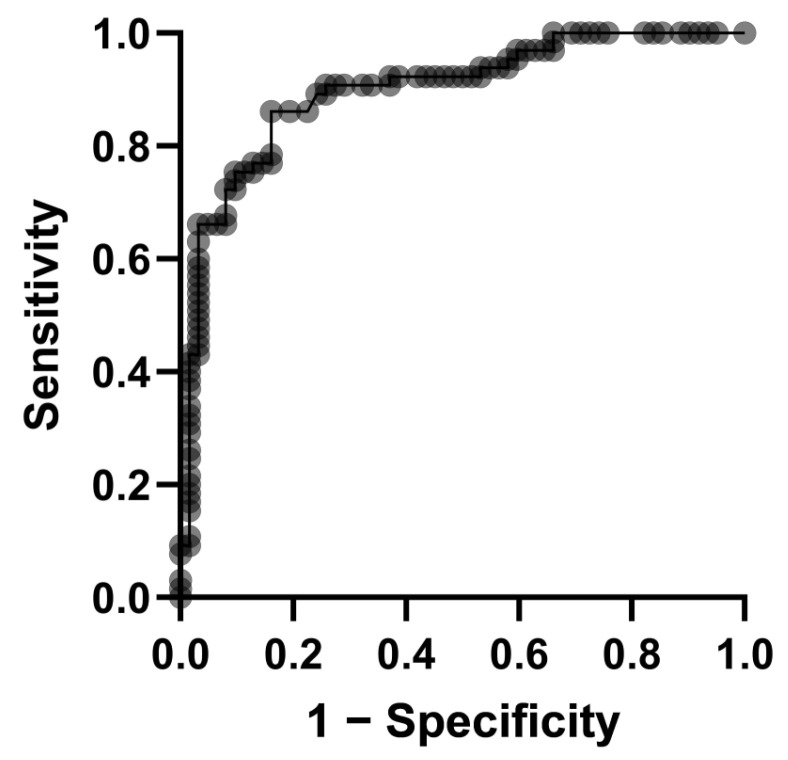
Receiver operating characteristic (ROC) curve of the multivariable logistic regression model evaluating factors associated with surgical management. The model demonstrated good discriminative performance with an area under the curve (AUC) of 0.89 (95% CI 0.84–0.95, *p* < 0.001).

In multivariable logistic regression analysis (Table 4), duration of symptoms remained independently associated with surgical management, with longer symptom duration associated with increased odds of surgery (OR 1.04 per month, 95% CI 1.02–1.06, *p* < 0.001). Purulent discharge was also strongly associated with surgical management, although the wide confidence interval suggests limited precision of the estimate. ENT comorbidity was associated with surgical management in the multivariable model, although the direction of this association differed from univariate analysis (OR 4.98, 95% CI 1.77–16.33, *p* = 0.002). In contrast, dry eye syndrome, fluorescein dye disappearance score, and history of ocular trauma were not independently associated with surgical management. The model demonstrated good discriminative performance, with an area under the receiver operating characteristic curve (AUC) of 0.89 (95% CI 0.84–0.95, *p* < 0.001) (Figure 3). No severe multicollinearity was identified among variables included in the multivariable model. Variance inflation factor (VIF) values were 2.17 for duration of symptoms, 2.47 for purulent discharge, 5.07 for history of ocular trauma, 1.47 for dry eye syndrome, 3.77 for fluorescein dye disappearance score, and 2.30 for ENT comorbidity. However, the significant Hosmer–Lemeshow test and wide confidence intervals for certain variables indicate that the regression model should be interpreted cautiously despite its good discriminative performance.

## 4. Discussion

Importantly, this study focuses on factors associated with surgical decision-making rather than imaging characteristics alone. In this study, CT-DCG findings provided anatomical context for the clinical presentation of chronic epiphora. Distal nasolacrimal duct involvement, including stenosis and obstruction, was the most common finding, which is consistent with the known predominance of PANDO in adult populations [7]. The observed female predominance in the study cohort further aligns with previous reports, which have attributed this distribution to anatomical and hormonal factors affecting the lacrimal drainage system [5]. The distribution of CT-DCG findings in this cohort highlights the potential of this modality to identify both distal and proximal causes of lacrimal obstruction, as well as associated inflammatory conditions such as dacryocystitis and canaliculitis. These observations are consistent with previous reports suggesting that CT-DCG can provide detailed anatomical information of the lacrimal drainage system beyond conventional irrigation or probing [8]. In particular, the ability to differentiate between complete obstruction, partial stenosis, and alternative causes such as punctal abnormalities may have implications for treatment planning [12,13]. Nevertheless, certain imaging-related findings, particularly punctal eversion, demonstrated significant associations with surgical management in univariate analysis and may warrant further investigation in larger cohorts.

Among clinical variables, purulent discharge was strongly associated with surgical management. This finding likely reflects its role as a clinical marker of infection or advanced obstruction of the lacrimal drainage system [7]. Similarly, longer duration of symptoms was associated with an increased likelihood of surgical intervention, suggesting that chronicity may be linked to more persistent or structural abnormalities that are less responsive to conservative management. These observations are consistent with the clinical understanding that prolonged disease duration and infectious signs may indicate a higher probability of irreversible obstruction [14,15]. In contrast, functional parameters, including the fluorescein dye disappearance test and the presence of dry eye syndrome, were not independently associated with surgical management after adjustment for other variables. This may suggest that functional assessments alone are insufficient to distinguish patients who require surgical intervention from those who may benefit from conservative management [10]. However, these tests remain clinically relevant as part of the overall diagnostic evaluation and may still provide useful complementary information [16].

Importantly, surgical management in this study reflected real-world clinical decision-making based on combined clinical, functional, and imaging findings rather than an independent measure of confirmed surgical necessity. Therefore, the regression model should be interpreted as identifying factors associated with surgical decision-making within this cohort rather than predicting definitive need for surgery or postoperative outcomes.

The association between ENT comorbidity and surgical management differed between univariate and multivariable analyses, suggesting potential confounding or interaction with other clinical variables. Multicollinearity analysis did not demonstrate severe collinearity among variables included in the regression model. Nevertheless, the observed change in direction of association may reflect the complexity of clinical decision-making, residual confounding, or model instability related to the relatively limited sample size, and should therefore be interpreted cautiously. Further studies with larger cohorts are needed to clarify this relationship. From a clinical perspective, CT-DCG may serve as an adjunctive imaging modality in the evaluation of selected patients with chronic epiphora by providing complementary anatomical information regarding the level and extent of lacrimal drainage abnormalities. Although several clinical features, particularly prolonged symptom duration and purulent discharge, were strongly associated with surgical management, imaging may still assist in distinguishing structural from functional causes of epiphora and support surgical planning in diagnostically complex or equivocal cases. Therefore, the role of CT-DCG in this study was not to replace standard clinical evaluation, but rather to supplement ophthalmologic assessment with detailed anatomical characterization. The potential benefits of CT-DCG should also be balanced against radiation exposure, and its use may therefore be most appropriate in selected patients with diagnostically uncertain or complex presentations. At the same time, the lack of independent association for certain functional tests highlights the importance of a multimodal diagnostic approach [17,18].

This study has several limitations. First, it was conducted at a single center, which may limit the generalizability of the findings. Second, the retrospective observational design does not allow assessment of causal relationships and may be associated with information bias due to reliance on existing clinical records. Third, selection bias may be present, as CT-DCG was selectively performed in patients with clinically suspected involvement of the nasolacrimal drainage system. This preselection may have enriched the study population for structural lacrimal pathology and could limit the generalizability of the findings to broader populations with epiphora. Finally, some clinical assessments, including grading of functional tests, may be subject to interobserver variability. Although the regression model demonstrated good discriminative performance, the significant Hosmer–Lemeshow test and wide confidence intervals for some variables, particularly purulent discharge, suggest limited model stability and possible overestimation of effect sizes related to the sample distribution. Therefore, these findings should be interpreted cautiously. In addition, postoperative outcomes and treatment response data were not available, limiting assessment of whether imaging-associated surgical decisions translated into improved clinical outcomes. In addition, advanced objective tear film assessment techniques such as anterior segment optical coherence tomography (AS-OCT) were not available in this study. The use of predominantly clinical and functional tests may therefore have introduced measurement variability.

In conclusion, clinical features, particularly purulent discharge and symptom duration, were associated with real-world surgical management decisions, while CT-DCG provided complementary anatomical information in patients with suspected nasolacrimal duct pathology. These findings support the role of CT-DCG as an adjunctive imaging modality in selected cases of chronic epiphora; however, further studies incorporating postoperative outcomes are needed to determine its impact on clinical benefit and long-term management.

## Figures and Tables

**Table 1:** Baseline characteristics of the study population.

Variable	Value
Age, years, median (IQR)	64 (49.5–70)
Female, *n* (%)	102 (80.3%)
Duration of symptoms, months, median (IQR)	26 (9.5–53)
Dry eye syndrome, *n* (%)	87 (68.5%)
Purulent discharge, *n* (%)	52 (40.9%)
ENT comorbidity, *n* (%)	57 (44.9%)
History of ocular trauma, *n* (%)	20 (15.7%)
Intermittent symptom exacerbation, *n* (%)	46 (36.2%)
Fluorescein dye disappearance score, median (IQR)	2 (2–3)
Schirmer test OD, mm, median (IQR)	12 (10–16)
Schirmer test OS, mm, median (IQR)	13 (11–16)

**Table 2:** Distribution of CT-DCG findings (*n* = 127).

CT-DCG Finding	*n* (%)
NLDS stenosis	50 (39.4%)
NLDS obstruction	35 (27.6%)
Dacryocystitis	21 (16.5%)
Punctal eversion	10 (7.9%)
Canaliculitis	6 (4.7%)
Dacryolith	3 (2.4%)
Dacryopericystitis	2 (1.6%)

**Table 3:** Comparison of clinical and imaging characteristics according to management strategy.

Variable	Surgery (*n* = 65)	Non-Surgical (*n* = 62)	*p*-Value
Age, years, median (IQR)	65 (52–70)	59.5 (47.5–66.8)	0.213
Sex (female), *n* (%)	54 (84.4%)	47 (75.8%)	0.381
Duration of symptoms, months, median (IQR)	38 (19–92)	14 (6–30)	<0.001
Dry eye syndrome, *n* (%)	52 (80.0%)	35 (56.5%)	0.007
Purulent discharge, *n* (%)	43 (66.2%)	9 (14.5%)	<0.001
ENT comorbidity, *n* (%)	21 (32.3%)	36 (58.1%)	0.004
History of ocular trauma, *n* (%)	12 (18.5%)	8 (12.9%)	0.999
Intermittent symptom exacerbation, *n* (%)	14 (21.5%)	24 (38.7%)	0.052
Fluorescein dye disappearance score, median (IQR)	3 (2–3)	2 (2–3)	<0.001
Schirmer test OD, mm, median (IQR)	13 (11–16)	12 (11–17)	0.378
Schirmer test OS, mm, median (IQR)	13 (11–15)	13 (12–16)	0.400
NLDS stenosis, *n* (%)	21 (32.3%)	27 (43.5%)	0.205
NLDS obstruction, *n* (%)	18 (27.7%)	16 (25.8%)	0.843
Dacryocystitis, *n* (%)	13 (20.0%)	9 (14.5%)	0.486
Punctal eversion, *n* (%)	9 (13.8%)	1 (1.6%)	0.017
Canaliculitis, *n* (%)	1 (1.5%)	6 (9.7%)	0.058
Dacryolith, *n* (%)	3 (4.6%)	0 (0%)	0.245
Dacryopericystitis, *n* (%)	0 (0%)	2 (3.2%)	0.236

**Table 4:** Multivariable logistic regression analysis of predictors of surgical management.

Variable	Adjusted OR	95% CI	*p*-Value
Duration of symptoms (per month)	1.04	1.02–1.06	<0.001
Purulent discharge	22.8	7.18–89.05	<0.001
ENT comorbidity	4.98	1.77–16.33	0.002
Dry eye syndrome	1.28	0.47–3.62	0.63
Fluorescein score	0.75	0.41–1.36	0.34
History of trauma	0.46	0.10–2.01	0.30

## Data Availability

The data presented in this study are available on request from the corresponding author. The data are not publicly available due to privacy and ethical restrictions.

## References

[1] Serbest Ceylanoğlu K., Ceylano S., Acar A. (2023). Overview of Epiphora Referred to Oculoplastic Surgery Clinic in Adults. Beyoglu Eye J..

[2] Tai E.L.M., Kueh Y.C., Abdullah B., Chang B. (2025). Interventions in functional epiphora–a systematic review. Int. J. Ophthalmol..

[3] Li R., Li Y., McArdle B. (2024). Approach to the watery eye. Aust. J. Gen. Pract..

[4] Jordan D., Mawn L., Anderson R.L. (2012). The Lacrimal System. Surgical Anatomy of the Ocular Adnexa.

[5] Jin H., Chen X., Liu Y. (2025). Upper eyelid predominance of meibomian gland dysfunction in postmenopausal women with primary acquired nasolacrimal duct obstruction. BMC Ophthalmol..

[6] Valcheva K.P., Murgova S.V. (2024). Primary acquired nasolacrimal duct obstruction—Epidemiology, clinical signs and surgical treatment. Folia Med..

[7] Ali M.J. (2023). Etiopathogenesis of primary acquired nasolacrimal duct obstruction (PANDO). Prog. Retin. Eye Res..

[8] Papathanassiou S., Koch T., Suhling M.C., Lenarz T., Durisin M., Stolle S.R.O., Raab P. (2019). Computed Tomography Versus Dacryocystography for the Evaluation of the Nasolacrimal Duct—A Study With 72 Patients. Laryngoscope Investig. Otolaryngol..

[9] Öncel Acır N., Cetinkaya S. (2025). The etiological spectrum of epiphora: Functional epiphora as an overlooked cause. Indian J. Ophthalmol..

[10] Usmani E., Shapira Y., Selva D. (2023). Functional epiphora: An under-reported entity. Int. Ophthalmol..

[11] Choi S.C., Lee S., Choi H.S., Jang J.W., Kim S.J., Lee J.H. (2016). Preoperative Computed Tomography Findings for Patients with Nasolacrimal Duct Obstruction or Stenosis. Korean J. Ophthalmol..

[12] Yang L., Yin Z., Sun H., Li H., Wang P., Wang Y., Zhang L., Li J., Zhao Y., Pan Y. (2025). The novel diagnostic value and characteristic imaging features of CT dacryocystography in primary lacrimal canaliculitis. Sci. Rep..

[13] Raghuwanshi S., Yadav N., Raghuwanshi S., Raghuwanshi S. (2021). Multi-Detector CT Instillation Dacryocystography and Its Role in the Diagnosis of Lacrimal Drainage System Blocks. Indian J. Otolaryngol. Head Neck Surg..

[14] Sáenz Araya D., Lizano Guevara F., Sevilla Torres E., Fernandez Vinocour D., Rojas Peláez A. (2025). Anatomical and Functional Alterations in Nasolacrimal Duct Obstruction: A Comprehensive Review. Cureus.

[15] Agarwal A., Naik M., Ali M.J., Bothra N. (2024). The role of CT-DCG in hardware—Associated secondary acquired lacrimal duct obstruction: SALDO update study-(SUP)-Paper III. Am. J. Ophthalmol. Case Rep..

[16] Craig J.P., Nichols K.K., Akpek E.K., Caffery B., Dua H.S., Joo C.-K., Liu Z., Nelson J.D., Nichols J.J., Tsubota K. (2017). TFOS DEWS II Definition and Classification Report. Ocul. Surf..

[17] Gupta N. (2021). Imaging in Disorders of the Lacrimal Drainage System. Endoscopic Dacryocystorhinostomy.

[18] Hamdy H., El-Anwar M.W., Mobasher M.A., Amin M.I., Abdelhamid H. (2025). Role of CT dacryocystography in the management of lacrimal drainage system obstruction. Egypt. J. Otolaryngol..

